# User demographics and real-world use of the digital diabetes companion app dibi: a retrospective analysis

**DOI:** 10.3389/fdgth.2026.1824377

**Published:** 2026-07-27

**Authors:** Patrizia Brunner, Steffeni Papukchieva, Benjamin Friedrich, Julia Steinmetz-Späh, Marie Hammer, Joachim Kienhöfer, Thomas Ebert, Anne Lautenbach

**Affiliations:** 1 Temedica GmbH, Munich, Germany; 2 Else Kroener Fresenius Center for Digital Health (EKFZ), Technical University Dresden, Dresden, Germany; 3 Novo Nordisk Pharma GmbH, Mainz, Germany; 4 Medical Department III – Endocrinology, Nephrology, Rheumatology, University of Leipzig Medical Center, Leipzig, Germany; 5 Ambulanzzentrum des UKE GmbH, Department of Endocrinology and Diabetology, University Obesity Centre, University Hospital Hamburg-Eppendorf, Hamburg, Germany

**Keywords:** diabetes, DIBI, digital health, real-wold data, treatment

## Abstract

**Background:**

Type 2 Diabetes (T2D) is a chronic condition requiring lifelong personalized management to prevent disease progression and complications. Mobile health applications like dibi can substantially support patients in daily disease management.

**Objective:**

This study analyzes user demographics, self-reported treatment characteristics, and early feature use among users of the dibi digital diabetes companion app to better understand app uptake and feature use, advance personalized care and facilitate predictive healthcare strategies.

**Methods:**

Of 5,744 dibi users, 2,422 (42.2%) provided consent and completed registration (date: 15.12.24). Users were included if they had active consent and were aged ≥18 years, resulting in 2,262 users for the main analysis. Users with missing or invalid entries were excluded from respective parameter-specific analysis. Among all included users, 1,253 (55.3%) defined at least one medication plan with overall 1,039 unique medications, and 514 (22.7%) users utilized the adherence feature at least once to track medication intake.

**Results:**

Of the included users, 89.4% were patients with T2D, mostly male (57.5%), aged 56–65 years (33.5%) and recently diagnosed (0–1 year since self-reported diagnosis). Female T2D users appeared to be distributed toward younger age groups than male users and more often chose lifestyle changes only during onboarding. Most T2D users (36.9%) reported either treatment with oral antidiabetics (OAD) only or lifestyle modifications alone (17.1%). Of the T2D patients who used medication plans, the majority (57.0%) reported using only OADs, while 7.2% reported only non-diabetes medications. More escalated treatment regimens were observed with longer disease duration. While 87.5% confirmed medication intake at least once, 29.6% used the adherence feature only once.

**Conclusion:**

This analysis demonstrates that the dibi app reached a predominantly T2D user population in Germany. It provides insights into treatment patterns and patient reported lifestyle changes. The dibi cohort reflects trends seen in other studies, representing real-world disease management. These findings indicate that inclusion of medical questionnaires, and clinical metrics, such as HbA1c, will deepen our understanding of the disease and enable treatment-lifestyle correlations.

## 1 Introduction

Type 2 Diabetes (T2D) is a highly prevalent, chronic metabolic disease, in which chronic energy imbalance, favor of nutrient-storage pathways, and other pathomechanisms contribute to insulin resistance, impaired insulin secretion, and subsequent hyperglycemia (1). The condition requires personalized and continuous management to prevent complications such as cardiovascular disease, neuropathy, and kidney failure (2). Reducing hyperglycemia is first and foremost attempted by adjusting lifestyle factors of affected patients. If this is not sufficient, numerous different antidiabetic drugs are available to reduce glycemic parameters, as well as the risk to suffer from cardiovascular complications. As the last treatment step, patients are recommended to use insulin injection therapy to adequately manage their glucose metabolism (3).

The daily management of T2D includes monitoring the nutritional intake, regular exercise and medication adherence, as well as routine glucose measurements and appointments with healthcare practitioners. The disease itself and its management significantly impair the quality of life of patients (4).

Mobile health applications have revolutionized diabetes management by offering patients real-time tracking of blood glucose levels, medication adherence, dietary patterns, and physical activity (5). These apps also provide feedback, reminders, and access to educational resources, empowering patients to self-manage their chronic condition and enhance their medication adherence as well as their glycemic control (6, 7). Companion apps, in particular, create an interactive ecosystem between patients and healthcare providers, facilitating personalized care. The wealth of data generated by these apps, including demographic information, opens new opportunities for research and public health insights. Crucially, such tools can only deliver clinical benefit if patients actually adopt them and continue to use them, yet real-world adoption and engagement are highly variable and remain poorly characterised for newly launched apps. Understanding who uses a given app, and how intensively, is therefore a prerequisite both for designing effective, tailored strategies to support sustained app use and for interpreting any downstream clinical signal.

Several digital tools are already available to patients with diabetes in Germany. These range from self-tracking or diary apps focused primarily on glucose and bolus logging (for example mySugr) to reimbursable digital health applications (Digitale Gesundheitsanwendungen, DiGA) that deliver structured behaviour-change programmes. In contrast to single-function trackers, dibi was designed as a T2D-tailored companion that combines onboarding-based capture of demographic and treatment information, medication and adherence tracking, and general lifestyle recommendations within one application, which is what enables the demographic and treatment-pattern analysis presented here. Where possible, we relate the data points that are directly comparable with prior real-world sources, namely the distribution of treatment groups and the time since diagnosis, to published German real-world data in the Discussion.

This study aims to describe the demographic and health-related characteristics of users of dibi, a recently launched novel patient companion app, which was specifically tailored to patients with T2D and their needs. As an initial analysis of this app, this study focuses on analyzing demographic data from users of dibi. This will give critical insights into app uptake and feature use and information supporting future strategies to advance personalized medicine. Furthermore, it supports the development of predictive analytics, helping healthcare providers anticipate patient needs and optimize long-term outcomes.

## 2 Methods

### 2.1 Study design

This study was designed as a retrospective, observational, cross-sectional descriptive analysis of anonymized real-world data collected from registered users of the dibi app between 01 July 2024 and 15 December 2024. The analysis aimed to describe user demographics, self-reported diabetes-related characteristics, treatment patterns, and medication-plan use. The study was based on routine app use and no hypothesis testing was performed.

### 2.2 Data source

Dibi is a smartphone app designed to assist individuals with T2D by providing support tailored to their specific needs. It allows users to track their medication usage over time and regularly answer medical questionnaires on their quality of life and treatment satisfaction. After registration and consent, users completed an onboarding process in which they provided demographic and health-related information, including diabetes type, year of diagnosis, and current treatment. Users could subsequently create a medication plan by selecting one or more medications, defining the dosing schedule, and setting reminders. For each scheduled medication intake, users could report whether the dose had been taken or missed; these entries formed the basis for the adherence-feature analysis. In addition, the app offers general recommendations for maintaining a healthier lifestyle, such as tips on nutrition and physical activity. Dibi was specifically developed for and targeted at patients with T2D; while onboarding records the self-reported diagnosis and a small number of users reported T1D or other diabetes types, the app and all analyses in this study focus on T2D. The lifestyle recommendations are general, educational and non-individualised guidance on nutrition and physical activity, consistent with the basic therapy (“Basistherapie”) recommended for all T2D patients by the German national treatment guideline. To our knowledge, this is the first published report describing the dibi app; no prior usability or validation studies have been published, and an earlier version of this work is available only as a preprint.

### 2.3 Study participants

Registered dibi users between 01 July 2024 and 15 December 2024 were considered for this study if they had provided consent for the scientific use of their demographic and health-related data. The main inclusion criteria were active consent for health data usage for scientific purposes and age ≥18 years. Completed registration was defined as successful account registration and completion of the onboarding step. This study was based on a retrospective analysis of aggregated, anonymized real-world app data from consenting users. Therefore, no separate ethics committee approval was obtained. Additional analysis-specific inclusion and exclusion criteria were applied for individual variables and are described in the respective analysis steps.

### 2.4 Data collection

Demographic and health data (year of birth, gender, diabetes diagnosis, year of diagnosis, current treatment) were collected during onboarding with the options to skip the answer (age, gender, year of diagnosis). Treatment options were multi-select and included: Lifestyle changes, Oral Antidiabetics, Injectable Antidiabetics (Not Insulin), Basal Insulin, Bolus Insulin. It should be noted that “Lifestyle changes” was a self-reported selection within this multi-select treatment question. During the analysis period the app did not capture structured, quantitative tracking of lifestyle behaviours (for example logged meals, steps or activity sessions); reported lifestyle figures therefore reflect users’ self-reported treatment choice and not objective behavioural data.

After onboarding, users could enter the specific medications they take (multiple, no limit) from any approved medication in Germany. They further could define a medication schedule and select whether they adhered to the scheduled medication intake or not.

Each event was recorded with a time stamp. All data was stored and processed in a General Data Protection Regulation (GDPR)-compliant manner. Using a customized ingestion API, data was anonymized and injected into a secure Data Lake infrastructure. These data were further structured and inserted into pre-structured tables in a PostgreSQL-Database. A reporting and data integration dashboard solution (Permea Dashboard) was used to access and visualize the data from the precalculated tables.

### 2.5 Data processing and analysis

Data processing and analysis were performed with Python (version 3.9) from pre-calculated tables and further processed based on individual analyses. User numbers between 1 and 5 were not reported due to data protection regulations and are instead indicated as ‘cohorts too small’. All analyses were descriptive (counts and percentages per subgroup); no inferential statistical tests were applied and no adjustment for confounding factors was performed. Accordingly, between-group comparisons are reported descriptively and should not be interpreted as statistically tested differences.

### 2.6 Onboarding

For each parameter analyzed, only users who responded and provided details for the corresponding parameter were considered. Users with missing (categorized as ‘unknown’) or invalid entries were excluded. Additional criteria specific for each parameter under investigation and their classification were as follows:

Age was calculated using the year of birth. For all age-related analysis, users between the ages of 18 and 99 were considered. They were further classified into 5 subgroups based on age: 18–35, 36–45, 46–55, 56–65 and >65 years.

To study gender distribution, users who selected either female, male or diverse in the onboarding process were analyzed.

For analysis involving two or more parameters, users were included only if valid information was available for all parameters required for that analysis (e.g., gender-based age distribution). No imputation of missing data was performed. This applies to all cases throughout the study, where two or more parameters were involved, unless mentioned otherwise.

Time since diagnosis was computed using the year of diagnosis. Based on the years since diagnosis, users were grouped into 5 categories: 0–1 year, 2–5 years, 6–10 years, 11–20 years, and >21 years. Users who selected a year of diagnosis prior to their year of birth were excluded.

For all aforementioned parameters, the total number of unique users and subgroup percentages were calculated. To analyze treatment with respect to other parameters, the percentage of each treatment group per parameter group was calculated.

### 2.7 Medication

All patients with medication entries were included. Selected, individual medications were classified into the following onboarding-related categories, based on their approval and known therapeutic use:

- Non-Diabetes specific medication (including for example pain medication)
- Oral Antidiabetics
- Injectable Antidiabetics (not Insulin)
- Basal insulin
- Bolus insulin

For all medications, the total number of unique users and subgroup percentages were calculated.

To analyze medication with respect to age, gender or time since Diabetes diagnosis, the total number of users within each group was calculated and the percentage of each medication group per time group was computed.

### 2.8 Adherence feature use

To address adherence specifically, patients could implement individual medication plans in dibi and set reminders as well as report whether they took their planned medication or not. When setting up the medication plans, users could select specific medications. As >1,000 unique medications were reported from 1,187 T2D users, neither analyzing the individual products nor grouping them into mode of action was possible. Therefore, we grouped them according to the onboarding groups with the addition of “Non-Diabetes Medication” and no lifestyle changes.

Onboarding had to be completed, while medication plans are optional to use. To analyze the usage of the adherence feature, the number of users using it at least once was calculated as proportion of all users who used the medication feature.

## 3 Results

### 3.1 Demographic characteristics

**Figure 1:**
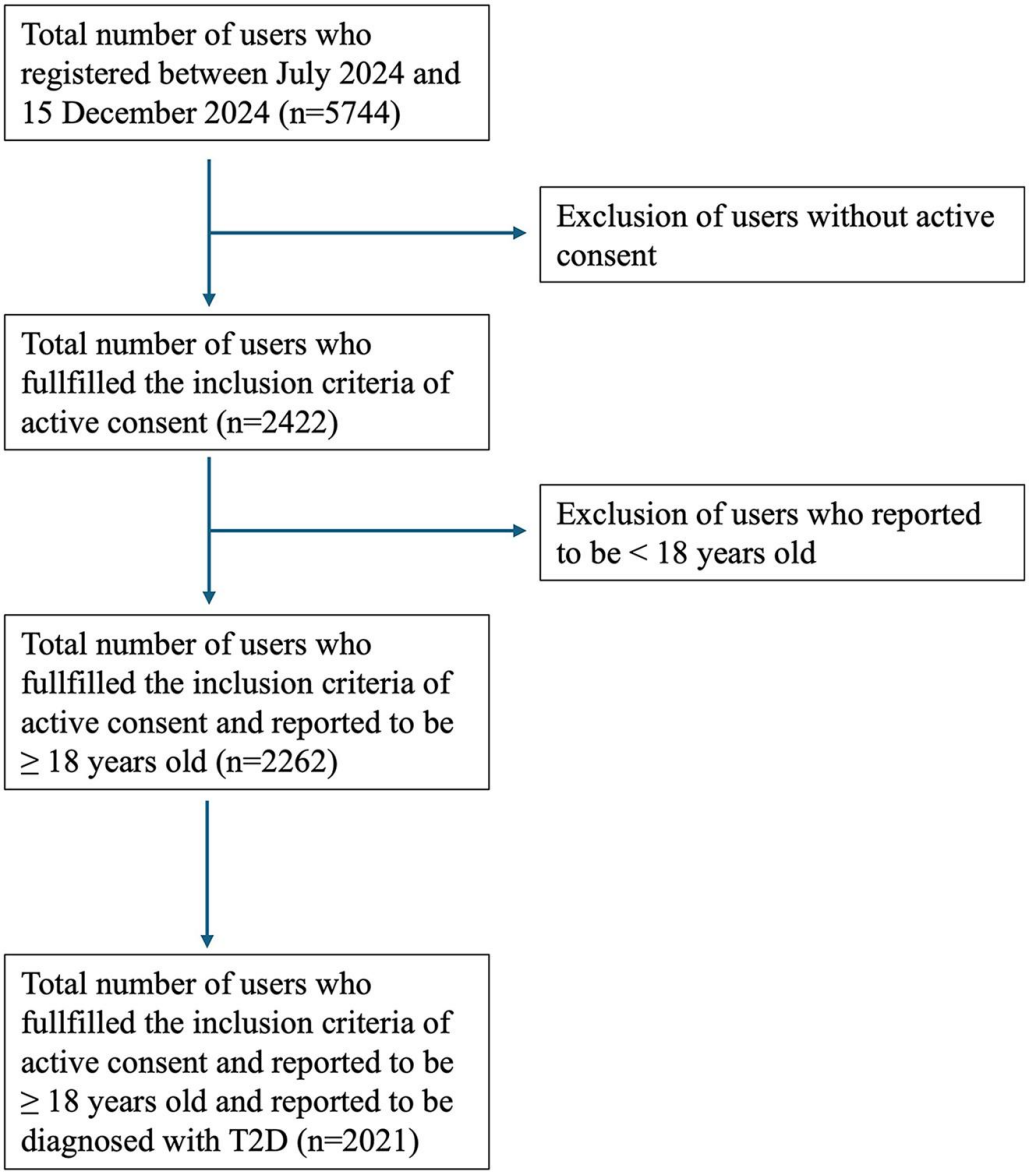
Overview of participant selection. T2D: Type 2 Diabetes.

Dibi was launched in July 2024 and a total of 5,744 users registered to use it until the timepoint of this data analysis. Of those 2,422 users provided consent and completed the registration. Data from 2,262 users, who fulfilled the inclusion criteria of active consent and reported to be ≥18 years old, were analyzed for this study (Figure 1). All of these users reported their diagnosis: 2,021 (89.4%) reported to be diagnosed with T2D, while 82 (3.6%) reported a Type 1 Diabetes diagnosis and 159 (7.0%) users selected another type (Diabetes related to malnutrition, Other specified diabetes, Unspecified diabetes). Detailed demographic data for all users are displayed in Table 1. As this patient companion was designed to target T2D patients, all further analyses only included the 2,021 T2D patients.

**Table 1:** Overview of study cohort according to categories ‘diagnosis’, ‘age group’, ‘gender’, ‘time since diagnosis’ and ‘current treatment’. For the multi-select treatment category, users could select more than one treatment option, therefore percentages may sum to more than 100%. User numbers between 1 and 5 were not reported due to data protection regulations and are instead indicated as ‘cohorts too small’.

Categories	Groups	Users	%
**Diagnosis**	Type 2 Diabetes	2,021	89.35
	Type 1 Diabetes	82	3.63
	Unspecified Diabetes	105	4.64
	Diabetes related to malnutrition	32	1.41
	Other specified diabetes	22	0.97
	**Total**	**2,262**	
**Age Group (years)**	18–35	101	4.47
	36–45	238	10.52
	46–55	523	23.12
	56–65	739	32.67
	>65	661	29.22
	**Total**	**2,262**	
**Gender**	Female	998	44.26
	Male	1,257	55.74
	Diverse	Cohort too small	
	**Total**	**2,255**	
**Time since diagnosis (years)**	0–1	647	30.26
	2–5	422	19.74
	6–10	344	16.09
	11–20	435	20.35
	>20	290	13.56
	**Total**	**2,138**	
**Current Treatment (multi select)**	Lifestyle changes	1,165	51.46
	Oral Antidiabetic (OAD)	1,181	52.16
	Injectable Antidiabetic (IAD)	306	13.52
	Basal Insulin	695	30.70
	Bolus insulin	389	17.18

**Figure 2:**
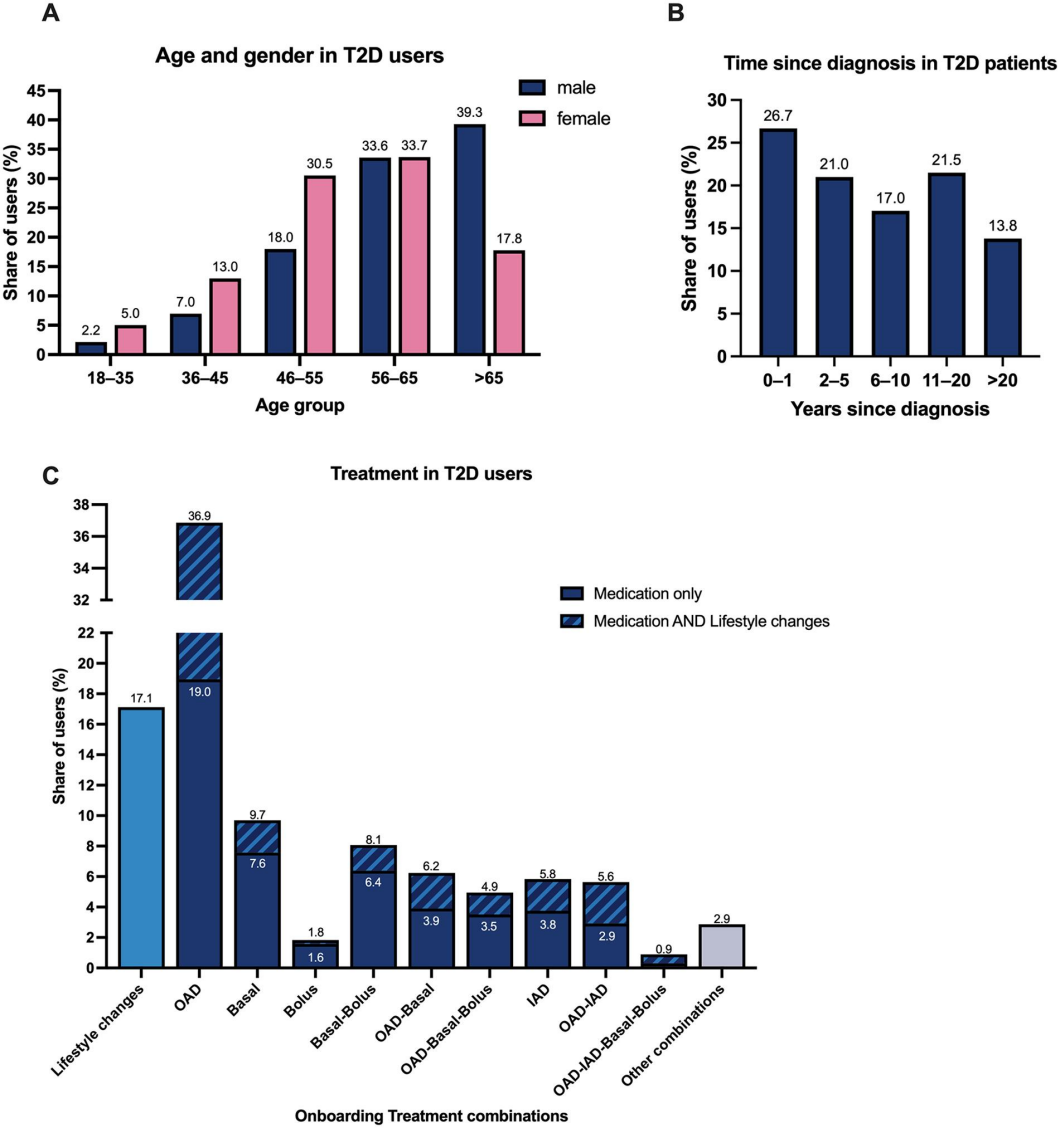
Age, gender **(A)**, time since diagnosis **(B)** and current treatment distribution **(C)** in T2D patients. Bar charts show share of users with exact proportions above each bar. A) As users were able to skip their gender, 2016 users who reported to be male or female were analyzed here. B) 99 patients skipped the time since diagnosis question and 2 users were excluded because of invalid answers (*n* = 1920). C) Based on the multi-select answer to the disease treatment different combinations were analyzed. Each medication treatment was further divided in medication only or, if the user selected it, medication and lifestyle adaptation. OAD, oral antidiabetic; IAD, injectable antidiabetic (Not Insulin).

Of the T2D patients, 1,161 (57.5%) were male and the largest share of users (678, 33.5%) was 56–65 years old. Female users appeared to be younger than male users (Figure 2A) and the highest share of users has been diagnosed with T2D for 0–1 years (512, 26.7%; Figure 2B). The distribution of different treatment combinations is shown in Figure 2C. The largest share of users reported current treatment with oral antidiabetics (OAD) with or without lifestyle adaptations (745, 36.9%). The second largest share reported to only implement lifestyle adjustments without any medication (346, 17.1%). Interestingly, in all medication groups only half of the users or less selected additional lifestyle adjustments, as would be recommended by treating guidelines. From the analyzed combinations, the smallest share reported taking all possible medication categories combined (0.9%).

### 3.2 Treatment differences

**Figure 3:**
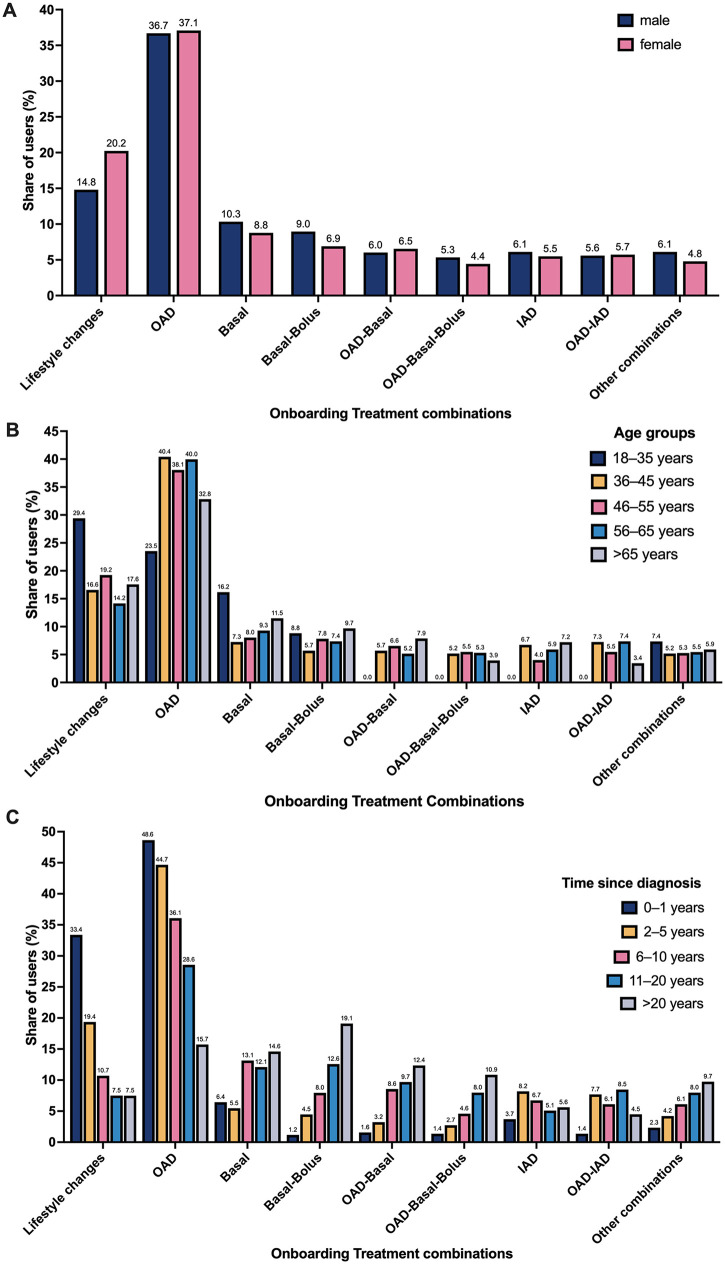
Different selected treatment combinations over gender **(A)**, age group **(B)** and time since diagnosis group **(C)** in T2D patients. Bar charts show share of users with exact proportions above each bar. Based on the multi-select answer to the disease treatment different combinations were analyzed. ‘Other combinations’ includes all not displayed combinations such as ‘Bolus only’, ‘OAD-IAD-Basal’, etc. Medication treatments include users who selected the respective medication**(s)** with or without lifestyle changes. A) As users were able to skip their gender, 2016 users who reported to be male or female were analyzed here. B) All 2021 users were evaluated regarding their selected treatment and age group. The group 18-35 years included few patients and too small cohorts are depicted as 0. C) 99 patients skipped the time since diagnosis question and 2 users were excluded because of invalid answers (*n* = 1920). OAD, oral antidiabetic; IAD, injectable antidiabetic (Not Insulin).

We further analyzed whether the reported treatment combinations differed in the respective age, gender and time since diagnosis subgroups (Figure 3). While male and female users reported similar medication combinations, a higher proportion of female users compared to men reported lifestyle changes only (20.2% vs. 14.8%; Figure 3A). The distributions of reported treatment combinations across age groups were broadly similar (Figure 3B). The user group 18–35 years included few users overall (*n* = 68), which biases the proportional distribution of their reported treatment combinations. Older users (>65 years old) reported more escalated treatment combinations, such as Basal-Bolus or OAD-Basal, and less OAD only treatment compared to younger users (36–65 years). Stratification by disease duration showed a descriptive pattern consistent with treatment escalation (Figure 3C). Users with a longer duration since T2D diagnosis were less likely to report lifestyle changes only or OAD-only treatment and more likely to report insulin treatment and combination treatments (e.g., OAD-Basal-Bolus). This trend was not observed for injectable antidiabetics (IAD) combinations.

### 3.3 Reported medications and adherence

**Figure 4:**
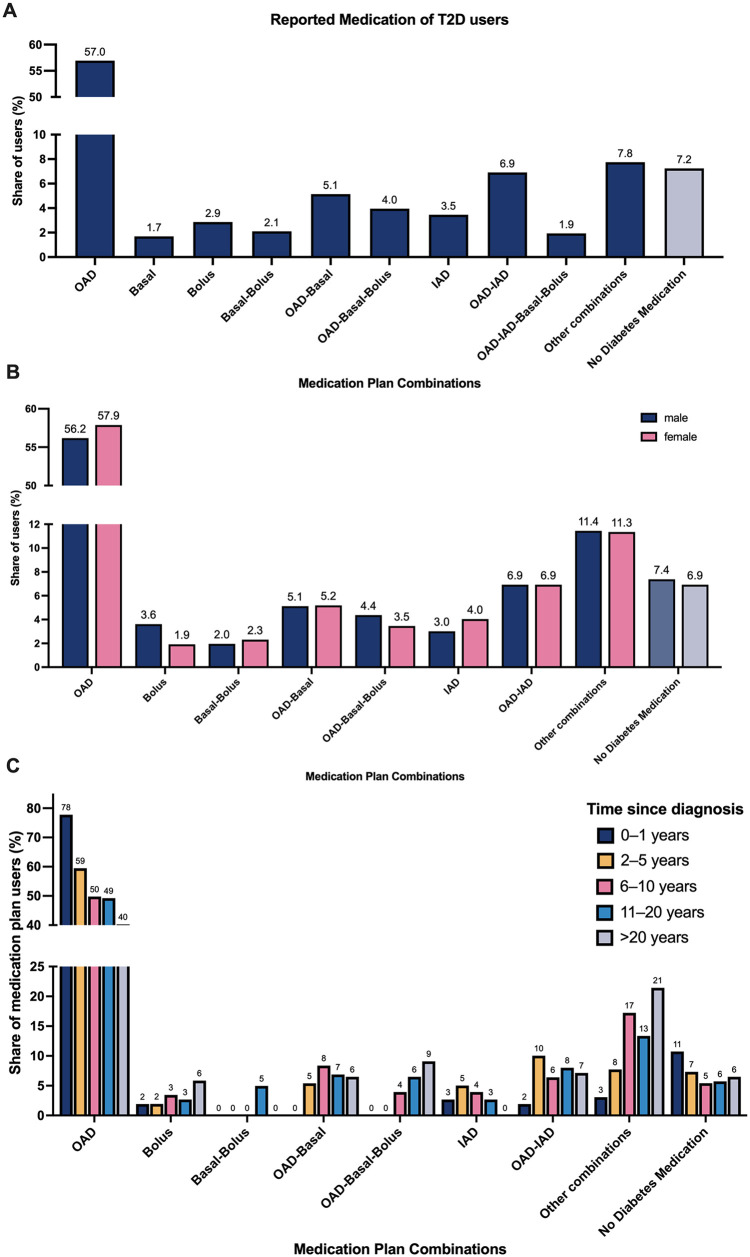
Different selected medication combinations over all T2D patients who filled out ≥1 medication plan **(A)**, gender **(B)** and time since diagnosis group **(C)** Bar charts show share of users with exact proportions above each bar. Based on the multitude of possible medication plans different combinations correlating to the treatment combinations from onboarding were analyzed. ‘Other combinations’ includes all not displayed combinations such as ‘Basal only’, ‘OAD-IAD-Basal’, etc. ‘No Diabetes medication’ includes all medications who are not approved for treatment of Diabetes. A) 1,187 T2D users filled out at least one medication plan and are included in this analysis. B) 1,184 T2D users, who filled out at least one medication plan reported to be either male or female. C) 1,139 users, who filled out as least one medication plan reported a valid diagnosis date. Different medication combinations included few patients and too small cohorts are depicted as 0. OAD, oral antidiabetic; IAD, injectable antidiabetic (Not Insulin);.

Of all T2D users, 1,187 patients defined at least one medication plan. The reported medications predominantly included single OADs (57%, Figure 4A), but the second largest group was non-antidiabetic medication, including pain killers or vitamins (7.2%). Patients reported their Bolus medication more often in the medication planner than during the onboarding, which can be explained by the need for documenting the dosing schema and frequent administration of bolus injections (2.9%). The reported medications were broadly similar between male and female users (Figure 4B). Interestingly, the escalation treatment was again visible in different time since diagnosis groups (Figure 4C). Users with longer disease progressions reported more prandial insulin medications and less single OAD medications, while users with less time since diagnosis reported more single OAD medicine. IAD use and non-Diabetes related medication appeared comparable across the time since diagnosis groups.

Overall, 514 users (22.7% of all included users) utilized the adherence feature, and of those 29.6% recorded only one adherence-feature interaction, while 15.7% reported their adherence ≥10 times. The majority of patients who used the adherence feature confirmed at least one scheduled medication intake as taken, whereas 24% reported that they did not take their medication at least once. Deeper, longitudinal or mode-of-action-stratified adherence analyses were not feasible: the data reflect a single time point shortly after launch, and the resulting subgroup cell sizes were too small for meaningful analysis. The app did not collect clinical glycemic parameters (such as HbA1c or glucose values) during the analysis period, so the relationship between adherence and glycemic control could not be assessed. User numbers in different medication groups were too little for further (longitudinal) adherence analysis.

## 4 Discussion

In this observational study, we aimed to describe the demographic and health-related characteristics of dibi app users. According to onboarding data, the app was used predominantly by male and middle-aged individuals, with 56–65 years being the largest age group. While the majority of users had T2D, a smaller number suffered from T1D or other forms of diabetes. The largest subgroup had been diagnosed recently (defined as 0–1 years since self-reported diagnosis). The treatments and medications reported were diverse, ranging from unmedicated lifestyle changes to a combination therapy of OADs, Basal and Bolus Insulin as well as IADs. The use of more advanced treatments, such as insulin injections and medication combinations, increased with the duration since diagnosis, while the frequency of IAD use remained stable.

### 4.1 Demographics

Over all reported factors, the demographics of the users of our patient companion dibi are slightly younger and more often female when compared to the general T2D population in Germany (8–10). This was expected as research indicates that female patients are more likely to proactively seek out support apps compared to male patients (11, 12). This trend is observed across various health contexts, including online health communities, social network sites, and specific health conditions. While it is also known that digital companions are less likely to be used by elderly patients based on technological skills, psychological factors and design-related barriers (13–15), the largest user cohort of dibi was between 56 and 65 years, which aligned with the high prevalence of T2D in the elderly.

As T2D is a chronic disease, which can be influenced by lifestyle associated behaviors, while T1D as an autoimmune disease cannot, this patient companion was designed to specifically support T2D patients. Notably, the vast majority of included users (89.4%) reported a diagnosis of T2D, aligning with the app's intended target population. 30% of users were diagnosed within the past year. This observation may reflect a particular interest or need among newly diagnosed patients for digital self-management tools, potentially due to increased motivation or educational needs at the onset of disease. Interestingly, the app did not attract only recently diagnosed patients, albeit they did represent the largest subcohort, but the time since diagnosis subgroups were quite largely distributed (Figure 2B) highlighting the need for such a patient companion even 20 + years after initial T2D diagnosis. This can be explained by the fact that T2D is a chronic condition that is not considered curable, and its management may become more challenging over time, particularly in the absence of rigorous nutritional, physical, and medical adherence (16). However, it should be noted that, especially in cases of early diagnosis, disease progression can often be delayed significantly through sustained lifestyle modifications, potentially postponing the need for medication (17).

In summary, the demographic characteristics of the T2D users analyzed in this study suggest that dibi has successfully reached its intended target population, particularly middle-aged and newly diagnosed individuals. However, the gender and age differences in user characteristics point to potential opportunities for tailored strategies to support uptake and personalized interventions within the digital health framework.

### 4.2 Comparison to real-world data

When comparing the treatment groups distribution to other real-world studies in Germany, similarities and discrepancies can be detected: Thus, the national diabetes surveillance report 2019 (18) states that in 2010 17.3% of T2D patients were untreated, 9.3% only intervened with lifestyle changes, 48.2% intervened with oral antidiabetics, 11.6% used only Insulin and 13.6% a combination of antidiabetics and insulin. A distinction between insulin and non-insulin injectable therapies was not made at that time, as the GLP-1 receptor agonists were only introduced to the market within the last two decades (19). Similarly, claims data from the KV region Nordrhein from 2020 showed that 30.8% of T2D patients were unmedicated, 47.5% received oral antidiabetics and 21,7% used insulin with or without antidiabetics (20).

In dibi, 17.1% of users reported only lifestyle therapy, a proportion that may include both early-stage T2D patients and those favoring non-pharmacological approaches. Importantly, the dibi onboarding was based on the national treatment guidelines, which recommend lifestyle modification (“Basistherapie”) for all T2D patients. Therefore, selecting no treatment at all was not an option, which may explain the lower apparent rate of non-medicated users compared to claims data (20). Additionally, patients actively managing their disease are more likely to seek help and engage with digital health tools, potentially leading to a selection bias toward more actively treated individuals (21). About 36.9% of patients reported only OAD medication, which is slightly lower compared to ∼48% observed in claims data (20). However, when added to IAD and OAD-IAD use, we also show 48.3%, which is similar. On the other hand, there are more insulin-medicated users in dibi than in other real-world studies. We report ∼20% insulin only and ∼15% insulin/antidiabetic usage, which again could be accounted for by the bias that insulin-using individuals proactively use health-apps to support their daily life. However, there are also other real-world analyses that demonstrated higher numbers, such as a claims analysis from 2013 to 2015, which showed that 9.6% of T2D patients initiated insulin therapy in Germany (22), thereby probably contributing to a higher overall treatment number. While another showed that 4.9% received GLP-1 receptor agonists in 2020 (23), which is comparable to the dibi user cohort on injectable antidiabetics, keeping in mind that GLP-RA were on a strong rise during the last years.

### 4.3 Treatment escalation and disease duration

The treatment pattern by time since diagnosis is consistent with the clinical rationale of treatment escalation. It should be emphasized, however, that this is a cross-sectional association that reflects the expected natural history of T2D and progressing disease duration rather than any effect of the app itself; no causal or app-attributable inference can be drawn from these descriptive data. Users with longer disease durations reported more frequent use of insulin and combination therapies, whereas recently diagnosed users were more likely to report lifestyle-only or OAD-only treatment (20). This trend is consistent with both disease pathophysiology and current treatment guidelines. However, it is important to note that insulin therapy is not exclusively a late-stage intervention. Some individuals may require early insulin initiation due to *β* cell dysfunction and consecutively impaired insulin secretion (24). Unfortunately, information on clinical parameters, such as ß-cell function, was not collected in the dibi study, limiting the ability to assess insulin initiation rationale in subgroups.

### 4.4 Lifestyle modification and adherence challenges

The most unexpected finding was the low frequency of reported lifestyle modifications. Importantly, fewer than half of the users in any pharmacological treatment group reported concurrent lifestyle adjustments, despite clinical guidelines strongly advocating for this combination. This finding could highlight a potential area for targeted education and intervention within the app. This represents a concerning gap in adherence to guideline-recommended care. Most studies have shown that applying nutritional recommendations and physical activity is generally low and vary dependent on education, employment status and other psycho-demographic factors (25–28). Digital tools such as dibi may help bridge this gap. Numerous studies have shown that digital interventions, particularly those offering educational content and reminders, can improve patient adherence and promote behavior change (5, 29–32). Therefore, the dibi platform has the potential to support lifestyle intervention adherence over time, especially as users engage more deeply with its features. It is important to note, however, that lifestyle modification in this analysis was captured only as a self-reported onboarding selection rather than through structured behavioural tracking; the present data therefore describe whether users reported pursuing lifestyle changes, not how these behaviours were actually performed. Structured tracking of nutrition and physical-activity behaviours is a planned direction for future development of the app and subsequent analyses.

### 4.5 Medication plans and adherence tracking

Approximately half of all included users completed a medication plan, which allowed detailed tracking of prescribed drugs, dosing, and timing. These plans were optional, unlike the mandatory onboarding process. Users may have chosen to log only selected medications, such as bolus insulin or multi-drug OAD regimens. This might explain the higher proportion of complex treatments among medication plan users.

The adherence feature, which enables patients to report missed or taken doses, was used by 22.7% of included users. While early usage metrics are promising, longitudinal data are needed to evaluate the feature's impact on sustained medication adherence. So far, this feature is particularly useful for the user and their daily disease management. In future analyses, it would be valuable to link reported treatments with clinical outcomes (e.g., HbA1c, hypoglycemia events, or symptom burden) and stratify by mode of action or treatment complexity.

## 5 Limitations

This study has several limitations. First, as dibi was launched recently, the analysis was based on a relatively small cohort and reflects data from a single time point, limiting the ability to assess trends or longitudinal effects. Second, the lack of additional clinical parameters, such as HbA1c values and data on co-morbidities, restricted the depth of analysis and precluded more nuanced correlations between demographic characteristics, disease burden and treatment patterns. Third, the reliance on self-reported data introduces potential for reporting bias or inaccuracies, including misclassification of treatments or underreporting of lifestyle interventions. Fourth, users who voluntarily download and register with a digital companion app are unlikely to be representative of the general T2D population, introducing a potential selection bias toward more actively engaged and digitally literate patients. Fifth, the analysis was purely descriptive and no inferential statistical testing was performed, so reported differences between subgroups should be interpreted as descriptive only. Finally, the findings have not been externally validated against an independent data source. These limitations should be considered when interpreting the findings.

## 6 Conclusion

We characterized the demographic profile of individuals with diabetes using the German digital health application dibi and assessed initial patterns of app usage. A detailed understanding of patient characteristics and their real-world behaviour provides valuable insights that can inform and enhance current treatment strategies, ultimately contributing to improved quality of life for people with T2D.

As a next step, we plan to investigate patient-reported outcomes related to quality of life, treatment satisfaction, and perceived disease severity. This will be conducted using standardized questionnaires and longitudinal data collected through the app, including HbA1c values and symptom tracking. These insights will support a more individualized and responsive approach to diabetes care.

## Data Availability

The original contributions presented in the study are included in the article/Supplementary Material, further inquiries can be directed to the corresponding author.

## References

[1] Roden M Shulman GI. The integrative biology of type 2 diabetes. Nature. (2019) 576(7785):51–60. 10.1038/s41586-019-1797-8 31802013

[2] Zheng Y Ley SH Hu FB. Global aetiology and epidemiology of type 2 diabetes mellitus and its complications. Nat Rev Endocrinol. (2018) 14(2):88–98. 10.1038/nrendo.2017.151 29219149

[3] Landgraf R Aberle J Birkenfeld AL Gallwitz B Kellerer M Klein HH. Therapy of type 2 diabetes. Exp Clin Endocrinol Diabetes. (2024) 132(07):340–88. 10.1055/a-2166-6755 38599610

[4] Garg P Duggal N. Type 2 diabetes mellitus, its impact on quality of life and how the disease can be managed-a review. Obes Med. (2022) 35:100459. 10.1016/j.obmed.2022.100459

[5] Hakami AM Almutairi B Alanazi AS Alzahrani MA. Effect of Mobile apps on medication adherence of type 2 diabetes Mellitus: a systematic review of recent studies. Cureus. (2024) 16:e51791. 10.7759/cureus.51791 38192533 PMC10772302

[6] Eberle C Löhnert M Stichling S. Effectiveness of disease-specific mHealth apps in patients with diabetes Mellitus: scoping review. JMIR MHealth UHealth. (2021) 9(2):e23477. 10.2196/23477 33587045 PMC7920757

[7] Shrivastava TP Goswami S Gupta R Goyal RK. Mobile app interventions to improve medication adherence among type 2 diabetes Mellitus patients: a systematic review of clinical trials. J Diabetes Sci Technol. (2023) 17(2):458–66. 10.1177/19322968211060060 34861793 PMC10012382

[8] Schmidt C Reitzle L Dreß J Rommel A Ziese T Heidemann C. Prävalenz und inzidenz des dokumentierten diabetes mellitus – referenzauswertung für die diabetes-surveillance auf basis von daten aller gesetzlich krankenversicherten. Bundesgesundheitsblatt Gesundheitsforschung Gesundheitsschutz. (2020) 63(1):93–102. 10.1007/s00103-019-03068-9 31792553

[9] Lehner CT Eberl M Donnachie E Tanaka LF Schauberger G Schederecker F. Incidence trend of type 2 diabetes from 2012 to 2021 in Germany: an analysis of health claims data of 11 million statutorily insured people. Diabetologia. (2024) 67(6):1040–50. 10.1007/s00125-024-06113-8 38409438 PMC11058936

[10] Jacobs E Rathmann W Tönnies T Arendt D Marchowez M Veith L. Age at diagnosis of type 2 diabetes in Germany: a nationwide analysis based on claims data from 69 million people. Diabet Med. (2020) 37(10):1723–7. 10.1111/dme.14100 31390484

[11] Mahmood A Kedia S Wyant DK Ahn S Bhuyan SS. Use of mobile health applications for health-promoting behavior among individuals with chronic medical conditions. Digit Health. (2019) 5:2055207619882181. 10.1177/2055207619882181 31656632 PMC6791047

[12] Bol N Helberger N Weert JCM. Differences in mobile health app use: a source of new digital inequalities? Inf Soc. (2018) 34(3):183–93. 10.1080/01972243.2018.1438550

[13] Askari M Klaver NS Van Gestel TJ Van De Klundert J. Intention to use medical apps among older adults in The Netherlands: cross-sectional study. J Med Internet Res. (2020) 22(9):e18080. 10.2196/18080 32624465 PMC7501579

[14] Ahmad NA Mat Ludin AF Shahar S Mohd Noah SA Mohd Tohit N. Willingness, perceived barriers and motivators in adopting mobile applications for health-related interventions among older adults: a scoping review. BMJ Open. (2022) 12(3):e054561. 10.1136/bmjopen-2021-054561 35264349 PMC8915330

[15] Pywell J Vijaykumar S Dodd A Coventry L. Barriers to older adults’ uptake of mobile-based mental health interventions. Digit Health. (2020) 6:2055207620905422. 10.1177/2055207620905422 32110429 PMC7016304

[16] Ahmad E Lim S Lamptey R Webb DR Davies MJ. Type 2 diabetes. Lancet. (2022) 400(10365):1803–20. 10.1016/S0140-6736(22)01655-5 36332637

[17] Look AHEAD Research Group; Wing RR. Long term effects of a lifestyle intervention on weight and cardiovascular risk factors in individuals with type 2 diabetes: four year results of the Look AHEAD trial. Arch Intern Med. (2010) 170(17):1566–75. 10.1001/archinternmed.2010.334. PubMed PMID: 20876408; PubMed Central PMCID: PMC3084497. 20876408 PMC3084497

[18] Robert Koch-Institut. National Diabetes Surveillance Report 2019. Berlin; (2019). p. 96. Report No. Available online at: https://diabsurv.rki.de/SharedDocs/downloads/DE/DiabSurv/diabetes-report_2019_eng.pdf?__blob=publicationFile&v=6 (Accessed January 01, 2026).

[19] Alfaris N Waldrop S Johnson V Boaventura B Kendrick K Stanford FC. GLP-1 single, dual, and triple receptor agonists for treating type 2 diabetes and obesity: a narrative review. eClinicalMed. (2024) 75:102782. 10.1016/j.eclinm.2024.102782 PMC11402415 39281096

[20] Arzneimittelkommission Der Deutschen Apotheker (AMK), Arzneimittelkommission Der Deutschen Ärzteschaft (AkdÄ), Bundesarbeitsgemeinschaft Selbsthilfe E. V. (BAG Selbsthilfe), Deutsche Dermatologische Gesellschaft E. V. (DDG), Deutsche Diabetes Gesellschaft E. V. (DDG), Deutsche Gesellschaft Der Plastischen RUÄCEV (DGPRAEC), NVL Typ-2-Diabetes - Langfassung [Text/pdf]. Bundesärztekammer (BÄK); Kassenärztliche Bundesvereinigung (KBV); Arbeitsgemeinschaft der Wissenschaftlichen Medizinischen Fachgesellschaften (AWMF). (2023). Available online at: 10.6101/AZQ/000503 (Accessed 2025 February 16)

[21] Van Smoorenburg AN Hertroijs DFL Dekkers T Elissen AMJ Melles M. Patients’ perspective on self-management: type 2 diabetes in daily life. BMC Health Serv Res. (2019) 19(1):605. 10.1186/s12913-019-4384-7 31462220 PMC6714441

[22] Wilke T Picker N Mueller S Geier S Foersch J Aberle J. Real-world insulin therapy in German type 2 diabetes mellitus patients: patient characteristics, treatment patterns, and insulin dosage. Diabetes Metab Syndr Obes Targets Ther. (2019) 12:1225–37. 10.2147/DMSO.S214288 PMC6664320 31440070

[23] Hennies N Görgens SW Killer J Otto T Richter LM Müller-Wieland D. Prevalence of obesity and cardiovascular disease in adults with type 2 diabetes and use of diabetes medication in Germany: a claims data study. Diabetes Obes Metab. (2024) 26(12):5636–45. 10.1111/dom.15931 39300717

[24] Ahlqvist E Storm P Käräjämäki A Martinell M Dorkhan M Carlsson A. Novel subgroups of adult-onset diabetes and their association with outcomes: a data-driven cluster analysis of six variables. Lancet Diabetes Endocrinol. (2018) 6(5):361–9. 10.1016/S2213-8587(18)30051-2 PubMed PMID: 29503172. 29503172

[25] MacDonald CS Ried-Larsen M Soleimani J Alsawas M Lieberman DE Ismail AS. A systematic review of adherence to physical activity interventions in individuals with type 2 diabetes. Diabetes Metab Res Rev. (2021) 37(8):e3444. 10.1002/dmrr.3444 33769660

[26] Katsaridis S Grammatikopoulou MG Gkiouras K Tzimos C Papageorgiou ST Markaki AG. Low reported adherence to the 2019 American diabetes association nutrition recommendations among patients with type 2 diabetes Mellitus, indicating the need for improved nutrition education and diet care. Nutrients. (2020) 12(11):3516. 10.3390/nu12113516 33203138 PMC7696891

[27] Krzemińska S Lomper K Chudiak A Ausili D Uchmanowicz I. The association of the level of self-care on adherence to treatment in patients diagnosed with type 2 diabetes. Acta Diabetol. (2021) 58(4):437–45. 10.1007/s00592-020-01628-z 33251559 PMC8053648

[28] Klinovszky A Kiss IM Papp-Zipernovszky O Lengyel C Buzás N. Associations of different adherences in patients with type 2 diabetes mellitus. Patient Prefer Adherence. (2019) 13:395–407. 10.2147/PPA.S187080 30936685 PMC6422420

[29] Volpi SS Biduski D Bellei EA Tefili D McCleary L Alves ALS. Using a mobile health app to improve patients’ adherence to hypertension treatment: a non-randomized clinical trial. PeerJ. (2021) 9:e11491. 10.7717/peerj.11491 34123593 PMC8166239

[30] Pérez-Jover V Sala-González M Guilabert M Mira JJ. Mobile apps for increasing treatment adherence: systematic review. J Med Internet Res. (2019) 21(6):e12505. 10.2196/12505 31215517 PMC6604503

[31] Armitage LC Kassavou A Sutton S. Do mobile device apps designed to support medication adherence demonstrate efficacy? A systematic review of randomised controlled trials, with meta-analysis. BMJ Open. (2020) 10(1):e032045. 10.1136/bmjopen-2019-032045 32005778 PMC7045248

[32] Al-Arkee S Mason J Lane DA Fabritz L Chua W Haque MS. Mobile apps to improve medication adherence in cardiovascular disease: systematic review and meta-analysis. J Med Internet Res. (2021) 23(5):e24190. 10.2196/24190 34032583 PMC8188316

